# Supplementary material for: GLI transcriptional repression regulates tissue-specific enhancer activity in response to Hedgehog signaling
Source: eLife. 2020 Jan 28;9:e50670. doi: 10.7554/eLife.50670 (PMC6986877; doi:10.7554/eLife.50670)
Supplement: Figure 1—source data 4. — Table showing the top 20 motifs uncovered from de novo motif analysis on HH-responsive GBRs. The enrichment is relative to matched genomic controls. Note that ‘HH_resp_2’ is the only motif with an enrichment value of greater than two and corresponds with a known GLI binding motif. [file elife-50670-fig1-data4.pdf]

Figure1-Source Data4: Enrichment of de novo motifs in HH-responsive GBRs

Motif ID	Logo	Enrichment
HH-resp_0.mat	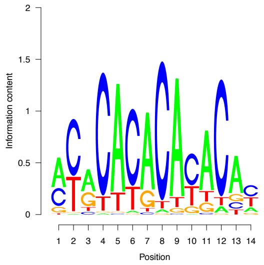	1.289071
HH-resp_1.mat	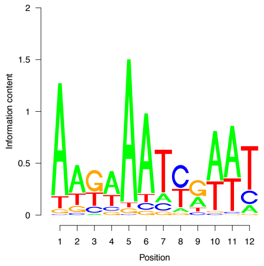	1.151352
HH-resp_2.mat (GLI)	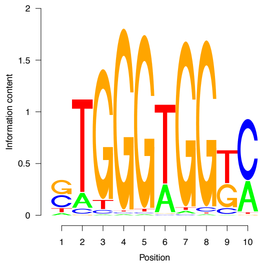	2.386835
HH-resp_3.mat	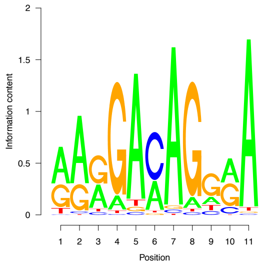	1.002307
HH-resp_4.mat	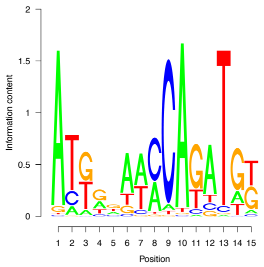	1.550095
