# Supplementary material for: GLI transcriptional repression regulates tissue-specific enhancer activity in response to Hedgehog signaling
Source: eLife. 2020 Jan 28;9:e50670. doi: 10.7554/eLife.50670 (PMC6986877; doi:10.7554/eLife.50670)
Supplement: Figure 2—source data 2. — The first column indicates genes previously identified as differentially expressed between Shh-/- and WT E10.5 limb buds (Lewandowski et al., 2015). The second column indicates the fold enrichment of H3K27me3 at the promoter compared to Input with the adjusted P-value indicated in the third column. The fourth column indicates whether the gene has a HH-dependent GBR (indicated by one and yellow shading) within the same presumptive TAD (Dixon et al., 2012). There are 22 HH-dependent target genes out of 80 HH-responsive genes. [file elife-50670-fig2-data2.pdf]

Gene	H3K27Me3_FC	Short_adjpval	HH-dependent target
Gli1	1.34490223	0.002457103	1
Mt1	1.36777668	0.002068091	0
Ptch2	1.0845207	0.591361833	1
Mt2	1.37889954	0.000563889	0
Fgf4	1.36514633	0.000514682	0
Sall1	1.50748216	3.81E-05	1
Fgf8	1.600302	5.21E-06	0
Slc30a1	0.96814871	0.929072999	0
Mamdc2	0.97024369	0.929072999	1
Ptch1	1.48290388	0.00011109	1
Hoxd12	1.7969164	3.07E-08	1
Ina	1.42003055	0.000131206	0
Mir124a-1hg	1.54781072	8.83E-06	0
Tubb3	1.3489779	0.002457103	0
Elavl3	1.38482142	0.001302466	0
Stmn2	1.1029505	0.529131937	0
Myt1	0.92698572	0.627708147	1
Ano1	0.97425689	0.929072999	0
Thy1	1.19988578	0.094851821	0
Gpnmb	1.19691299	0.156859893	0
Hsd11b2	0.97457349	0.929072999	0
Greb1	1.06487551	0.713614996	1
Hoxd13	1.55695651	1.08E-05	1
Nhlh2	1.20677635	0.094851821	0
Sall3	1.56665916	3.91E-05	0
Rfx4	1.68784771	3.27E-07	0
Nhlh1	0.96253975	0.909663337	0
Msi2	0.97613828	0.936366345	0
Msx3	1.33056839	0.001806362	0
Frem1	1.03743772	0.905527071	0
Robo3	1.52603201	1.52E-05	0
Cntn2	1.22373691	0.059298238	1
Kif1a	1.26492201	0.009168305	0
Aox3	1.01691291	0.955544074	0
Mtus1	1.20714392	0.100527233	1
Pou3f2	1.30711352	0.006055122	1
Fabp7	0.92104024	0.627708147	0
Neurod4	1.04620288	0.867697342	0
Enpp2	1.02202012	0.945464854	1
Grem1	1.15556618	0.229282288	1
Miat	1.1223053	0.325154174	0
Parp14	1.39479722	0.000848495	0
Car12	0.93955245	0.697658484	0

Rgs18	1.00838348	0.965607117	0
Sox2	1.40513276	0.000281732	1
Sost	1.17835263	0.204126463	0
Slc17a6	1.09351019	0.591361833	0
Kdm5d	1.12891816	0.634835736	0
Hoxd11	1.45990474	3.99E-05	1
Scrt2	1.39917125	0.000848495	0
Fibin	1.01332123	0.955544074	0
Chrna3	1.19279646	0.094851821	0
Chl1	1.0004852	0.995733302	0
Akap6	0.99519542	0.971681078	0
Epha3	1.01187877	0.955544074	0
Slc1a2	1.15025866	0.242629347	1
Srrm4	1.2161958	0.059298238	0
Mtmr11	0.97050852	0.929072999	0
Snord55	0.88604078	0.343358607	1
Cdk6	1.1884482	0.156859893	0
Acss2	1.06991533	0.686840634	0
Tfap2c	1.35243691	0.000848495	0
Ucp2	1.09326469	0.561706538	1
Scg3	0.9882618	0.955544074	0
Zic1	1.53793	3.76E-05	0
Hemgn	1.00712947	0.965607117	1
Ccnd1	1.16917452	0.244212035	0
Ppp2r2c	1.25145183	0.01523463	0
Pappa2	0.95490857	0.867697342	0
Apcdd1	1.01506751	0.955544074	0
Olig3	1.60708952	5.68E-06	0
Dock6	0.98955402	0.955544074	0
Gpr50	1.43701235	0.000514682	0
Adgre5	1.14922628	0.27683288	0
Lmo2	1.13289455	0.329980757	0
Lilrb4a	0.97122391	0.93422666	0
Elavl4	0.92294656	0.683227178	1
Krt15	1.10060766	0.591361833	0
Gja8	1.15535817	0.284854885	1
C3	1.06516189	0.721996352	0

Figure 2-Source Data 2. Hedgehog responsive genes with H3K27me3 enrichment. The first column indicates genes previously identified as differentially expressed between *Shh*$^{-/-}$ and WT E10.5 limb buds (Lewandowski et al. 2015). The second column indicates the fold enrichment of H3K27me3 at the promoter compared to Input with the adjusted P-value indicated in the third column. The fourth column indicates whether the gene has a HH-dependent GBR (indicated by 1 and yellow shading) within the same

presumptive TAD (Dixon et al. 2012). There are 22 HH-dependent target genes out of 80 HH-responsive genes.
